# Stable isotopes of C and N differ in their ability to reconstruct diets of cattle fed C_3_–C_4_ forage diets

**DOI:** 10.1038/s41598-022-21051-4

**Published:** 2022-10-13

**Authors:** David M. Jaramillo, Jose C. B. Dubeux, Martin Ruiz-Moreno, Nicolas DiLorenzo, Joao M. B. Vendramini, Lynn Sollenberger, Cheryl Mackowiak, Luana M. D. Queiroz, Daciele S. Abreu, Liza Garcia, Erick R. S. Santos, Burney A. Kieke

**Affiliations:** 1 Agronomy Department, North Florida Research and Education Center, University of Florida, 3925 Highway 71, Marianna, FL 32446 USA; 2 Department of Animal Sciences, North Florida Research and Education Center, University of Florida, 3925 Highway 71, Marianna, FL 32446 USA; 3 Agronomy Department, Range Cattle Research and Education Center, University of Florida, 3401 Experiment Station, Ona, FL 33865 USA; 4 Agronomy Department, University of Florida, 3111 McCarty Hall B, Gainesville, FL 32611 USA; 5 Soil and Water Sciences Department, North Florida Research and Education Center, University of Florida, 155 Research Rd., Quincy, FL 32351 USA; 6 Faculty of Agricultural, Life, and Environmental Sci-Ag, Food & Nutrition Science Department, University of Alberta, 16 St. and 85 Ave., Edmonton, AB T6G 2R3 Canada; 7 Marshfield Clinic Research Institute, 1000 N. Oak Ave., Marshfield, WI 54449 USA; 8 Present Address: Institute for Environmentally Integrated Dairy Management, USDA-ARS U.S. Dairy Forage Research Center, 2615 Yellowstone Dr., Marshfield, WI 54449 USA

**Keywords:** Plant sciences, Agroecology, Grassland ecology, Stable isotope analysis, Stable isotope analysis

## Abstract

Stable isotopes are useful for estimating livestock diet selection. The objective was to compare δ^13^C and δ^15^N to estimate diet proportion of C_3_–C_4_ forages when steers (*Bos* spp.) were fed quantities of rhizoma peanut (*Arachis* *glabrata*; RP; C_3_) and bahiagrass (*Paspalum* *notatum*; C_4_).Treatments were proportions of RP with bahiagrass hay: 100% bahiagrass (0%RP); 25% RP + 75% bahiagrass (25%RP); 50% RP + 50% bahiagrass (50%RP); 75% RP + 25% bahiagrass (75%RP); and 100% RP (100% RP). Feces, plasma, red blood cell (RBC), and hair were collected at 8-days intervals, for 32 days. Two-pool mixing model was utilized to back-calculate the proportion of RP based on the sample and forage δ^13^C or δ^15^N. Feces showed changes using δ^13^C by 8 days, and adj. R^2^ between predicted and observed RP proportion was 0.81 by 8 days. Plasma, hair, and RBC required beyond 32-days to reach equilibrium, therefore were not useful predictors of diet composition during the study. Diets were best represented using fecal δ^13^C at both 8-days and 32-days. By 32-days, fecal δ^15^N showed promise (R^2^ = 0.71) for predicting diet composition in C_3_–C_4_ diets. Further studies are warranted to further corroborate fecal δ^15^N as a predictor of diet composition in cattle.

## Introduction

Livestock grazing grass-legume mixed pastures can construct their own diets by selecting different forage species and fulfilling their nutritional requirements with varying proportions of each^1^. Diet selection in grazing animals is a function of preference modified by opportunity^2^, thus, when multiple species are present in a pasture, the accurate representation of the animal’s diet is difficult to achieve through pasture sampling alone. Knowing the true diet composition of a grazing animal is an important tool for linking pasture canopy composition and animal diet selection, and diet selection to animal performance. However, accurate representation of grazing animal diet composition has been a challenge in grazing research, with methods often relying on invasive techniques (e.g., collection of extrusa through oesophageal fistulae) which, among others, have certain limitations, including number of animals required, surgical costs, and difficulty in handling extrusa^3^. Other techniques utilizing plant wax components (e.g. n-alkanes, or long-chain alcohols) have also been employed for estimating diet botanical composition but remain challenging to employ under grazing due to intensive labor requirements related to dosing n-alkanes or long-chain alcohols, and elevated-costs related to sample processing^4^.

Stable isotope techniques provide additional options for inferring diet botanical composition across several animal species^5,6^, and thus far, C and N techniques have been implemented for this purpose. Stable isotope composition is expressed in delta (δ) notation, which is the difference between the ratio of heavy isotope (e.g. ^13^C or ^15^N) to light isotope (e.g. ^12^C or ^14^N) in a given sample, to the same ratio of the standard (Pee Dee Belemnite for C, or air for N)^5^. The C isotope techniques are contingent upon differences in the accumulation of C isotope in plants of contrasting photosynthetic pathways ^6^. Legumes and temperate, cool-season grasses are C_3_ (Calvin pathway) plants, while warm season, tropical and sub-tropical grasses are C_4_ (dicarboxylic acid pathway) plants. The C_3_ pathway discriminates against ^13^C in favor of ^12^C to a greater extent than C_4_, resulting in a more depleted (more negative) δ^13^C composition for C_3_ plants compared to that of C_4_ plants ^5,7^. The range of δ^13^C of C_3_ plants is −35 to −20‰, and C_4_ plants from −16 to −9‰ ^7^.

Stable N isotope techniques have been largely utilized in ecological studies to establish trophic schemes since consumers generally increase their ^15^N (the heavy N isotope) composition by a factor of 3‰ compared with their diet source ^5^. This occurs, to an extent, because of faster loss of ^14^N than ^15^N in metabolism and excretion leaves consumers with enriched (more positive) δ^15^N composition^5^. However, in agronomic studies, δ^15^N is often used to quantify biological N_2_-fixation^8^. Plant δ^15^N composition generally ranges between −10 and 5‰, with depleted δ^15^N being indicative of biological N_2_-fixation processes^9^. Plants that undergo biological N_2_-fixation have δ^15^N resembling that of the atmosphere, 0‰, where plants reliant upon soil mineral N, exhibit δ^15^N resembling that of the soil, oftentimes above 2‰^8,10^. Legumes are well known for their symbiotic relationship with N_2_-fixing bacteria, but grasses have also demonstrated potential to associate with N_2_-fixing microorganisms^11–13^.

Thus far, the use of fecal δ^13^C has been an accurate and rapid method for estimating the diet botanical composition of livestock grazing C_3_–C_4_ binary pastures^6^. This technique is advantageous in providing an inference on effects of dietary proportion of C_3_ or C_4_ species on short-term intake with ease of sample collection, and minimal sample processing requirements ^6,14^; however, a comparison across various body samples and their response to both ^15^N and ^13^C has yet to be made for beef cattle. Understanding the differences in response across body samples, in relation to C and N stable isotope composition is important for evaluating methodologies that aid in understanding grazing animal diet composition. This study was designed to compare the use of ^15^N and ^13^C composition in blood plasma, red blood cells (RBC), hair, and feces to estimate the diet botanical composition of beef steers fed C_3_–C_4_ binary diets. These body samples were selected provided their universal ease of collection across grazing studies. The two hypotheses in the study were: (1) δ^13^C of feces, plasma, red blood cells, and hair will be useful to back-calculate the diet of steers consuming C_3_–C_4_ binary diets; and (2) that δ^13^C is a more accurate predictor than δ^15^N of diet composition from steers consuming C_3_–C_4_ binary diets.

## Results

### Chemical composition of the diet

Concentrations of dry matter (DM) were similar across the five diets, and the concentrations of organic matter (OM) appeared to decrease with increasing proportion of RP inclusion (Table 1). Crude protein concentration was least in 0% RP, which contained bahiagrass only, and greatest in 100% RP. The in vitro digestible OM (IVDOM) concentrations were also least in 0% RP, at 46.4%, and increased to 67.8% in 100% RP. As the proportion of RP increased in the diet, the δ^13^C became more depleted, ranging from −16.04 to −29.41‰, in 0% RP and 100% RP, respectively. The reverse was observed for δ^15^N, where 0% RP was more depleted (−0.24‰) than 100% RP (1.71‰), and the treatment diets became more enriched with increasing proportion of RP.

**Table 1:** Chemical composition of the five treatment diets offered to beef steers, each with increasing proportion of rhizoma peanut (RP) hay into bahiagrass hay.

	% Rhizoma peanut hay in bahiagrass hay				
	0% RP	25% RP	50% RP	75% RP	100%RP
DM, %	91 ± 0.02	92 ± 0.02	91 ± 0.01	91 ± 0.02	91 ± 0.02
OM, % DM	94 ± 0.01	93 ± 0.06	93 ± 0.06	92 ± 0.07	91 ± 0.07
CP, % DM	8.4 ± 0.018	9.0 ± 0.001	9.2 ± 0.003	11.1 ± 0.021	12.0 ± 0.007
IVDOM, %	46.4 ± 1.2	53.8 ± 1.4	56.5 ± 1.1	63.6 ± 1.8	67.8 ± 1.0
δ^13^C, ‰	−16.04 ± 0.36	−19.39 ± 0.75	−22.14 ± 0.83	−26.22 ± 0.55	−29.41 ± 0.50
δ^15^N, ‰	−0.24 ± 0.01	0.38 ± 0.08	0.52 ± 0.01	1.55 ± 0.04	1.71 ± 0.08

Means indicate average of 10 samples ± standard deviation.

*IVDOM* in vitro digestible organic matter, estimated utilizing the two-stage technique described by Moore and Mott ^41^.

### Stable isotope composition across sample type

#### Feces

**Figure 1:**
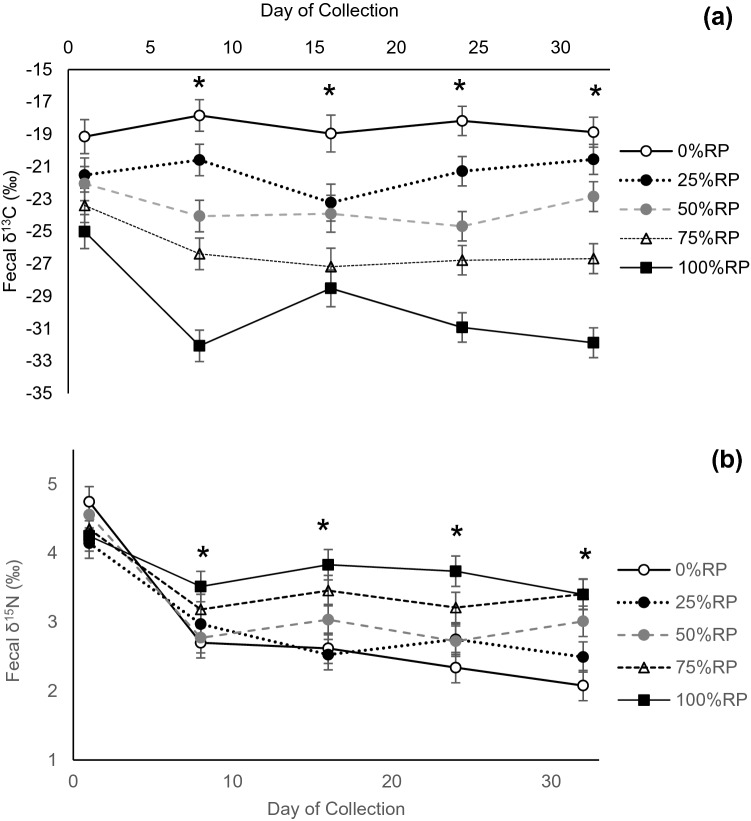
δ^13^C (**a**) and δ^15^N (**b**) feces of beef cattle consuming increasing proportions of rhizoma peanut (RP) in bahiagrass hay. Day of collection represents the number of days after dietary change. Asterisk indicates treatment differences (*P* ≤ 0.05) exist within the day of collection.

Throughout the experimental period, there was a treatment × collection day interaction on fecal δ^13^C (P = 0.002; Fig. 1). Feces from 0%RP remained at -18‰ throughout the 32-days experimental period, while 100%RP became the most depleted with time, reaching −31.8‰ by 32 days. The feces were depleted in their δ^13^C on the first day of collection, given the animals were fasted on 0 day, and therefore did not produce any fecal material upon collection. Despite depleted δ^13^C on 1 day, there were no treatment differences within this evaluation, whereas treatments within each evaluation day differed thereafter. The fecal δ^15^N composition also showed a treatment × collection day interaction (*P* = 0.005). Initial δ^15^N was 4.41‰ across all treatments, and by 32 days, 0%RP, 25%RP, and 50%RP were the most depleted, while 75%RP and 100%RP were the most enriched (Fig. 1). Across the five collection dates, there was a linear effect (*P* < 0.001) on the fecal δ^15^N composition, where the feces were more enriched with increasing proportions of RP, which might be indicative of discrimination against the lighter isotope, resulting in fractionation. Across all treatments, the feces became enriched in δ^15^N compared with the diets, and by 32 days, Δ (the fractionation factor, comparing diet and fecal δ^15^N) averaged 2.09‰ for each treatment.

#### Plasma

**Figure 2:**
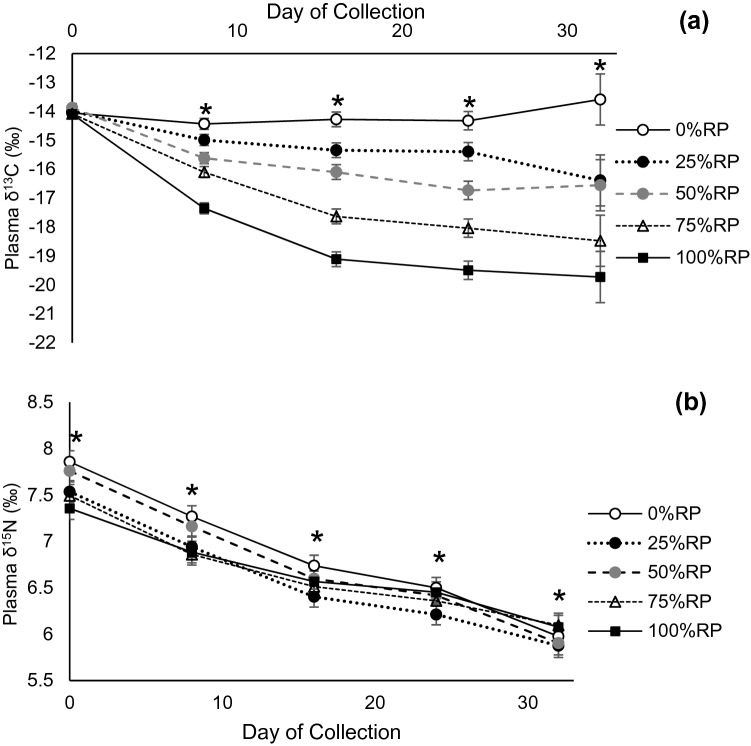
δ^13^C (**a**) and δ^15^N (**b**) plasma of beef cattle consuming increasing proportions of rhizoma peanut (RP) hay in bahiagrass hay. Day of collection represents the number of days after dietary change. Asterisk indicates treatment differences (*P* ≤ 0.05) exist within the day of collection.

Plasma δ^13^C composition became enriched as the proportion of RP increased in the diet. Furthermore, there was a treatment × collection day interaction (*P* < 0.001), in plasma δ^13^C. All treatments averaged −14.02‰ at the beginning (0 day) of the experimental period (Fig. 4). The 0%RP remained the most enriched, and was −14.32‰ by 32 days. In general, as the proportion of RP increased, the δ^13^C in the plasma became more depleted. The largest magnitude in difference from 0 to 32 days was in 100%RP, where the δ^13^C decreased from −14.09 to −19.73‰. By 16 days, plasma appeared to begin reaching equilibrium, since changes in δ^13^C were minimal in each of the treatments after 16 days. Estimating RP proportions based on plasma δ^13^C showed adjusted R^2^ of 0.82 and 0.74, for 8 and 32 days, respectively (Fig. 2). Similarly, the δ^15^N composition in the plasma also showed a treatment × collection day interaction (*P* = 0.049). Overall, there was a trend for plasma δ^15^N to become depleted throughout the duration of the experiment, where the plasma δ^15^N across all treatments was 7.60‰ at 0 day and was 5.99‰ by 32 days (Fig. 4).

#### Red blood cells

**Figure 3:**
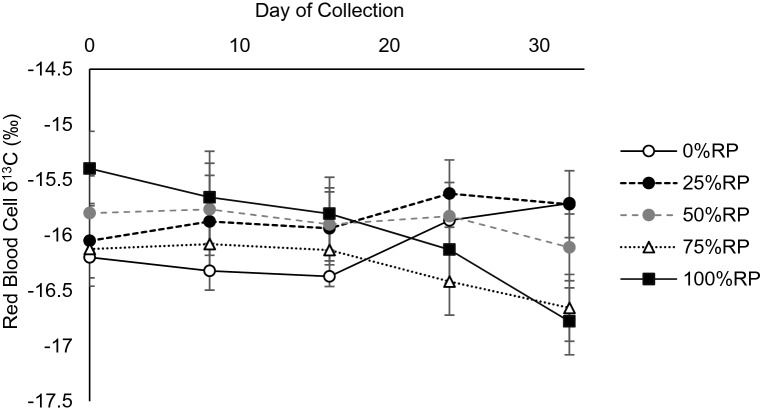
δ^13^C of red blood cells (RBC) of beef cattle consuming increasing proportions of rhizoma peanut (RP) in bahiagrass hay. Day of collection represents the number of days after dietary change.

Despite an observed treatment × collection day interaction on the δ^13^C composition of RBCs (*P* = 0.004; Fig. 3), there was no observable pattern to the response in terms of δ^13^C composition, nor was there an appearance of equilibrium by 32 days. Across all treatments, there was a difference of − 0.29‰ on δ^13^C of RBC, from 0 to 32 days, indicating small magnitude in change. Discrimination (Δ) was 13‰ in 100%RP, which was the largest among all treatments (*P* < 0.001). In addition, while there was a treatment effect (*P* = 0.04) on RBC δ^15^N composition (Table 2), a collection day effect was not observed (*P* = 0.55). The RBC δ^15^N decreased linearly (*P* = 0.004), as RP proportion increased in the diet (Table 2).

**Table 2:** δ^13^C and δ^15^N composition of red blood cells from beef steers consuming increasing proportions of rhizoma peanut (RP) hay in bahiagrass hay.

Diet type^A^	Red blood cells	
	δ^13^C (‰)	δ^15^N (‰)
0% RP	−16.09	6.26^a^
25% RP	−15.84	6.07^ab^
50% RP	−15.88	5.94^ab^
75% RP	−16.28	5.82^bc^
100% RP	−15.95	5.57^c^
SEM	0.309	0.142
*P* value	0.84	0.04
L^B^	0.87	0.004
Q^C^	0.85	0.68

Values are averages across five evaluations.

^A^Indicates percent (%) inclusion of rhizoma peanut (RP) hay into bahiagrass.

^B^L, indicates *P* value of linear polynomial contrast.

^C^Q, indicates *P* value of quadratic polynomial contrast.

^a–e^Means within a column followed by a common letter are not significantly different, according to LSD at the 5% significance level.

#### Hair

**Figure 4:**
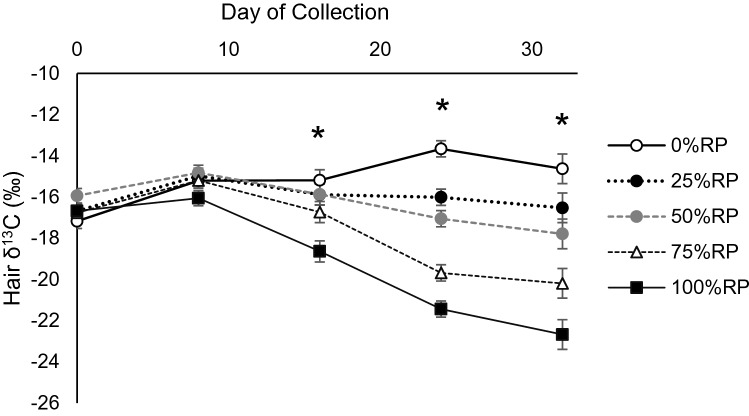
δ^13^C of hair from beef cattle consuming increasing proportions of rhizoma peanut (RP) in bahiagrass hay. Day of collection represents the number of days after dietary change. Asterisk indicates treatment differences (*P* ≤ 0.05) exist within the day of collection.

Hair δ^13^C composition showed a treatment × collection day interaction (*P* < 0.001; Fig. 4). During the 0-day and 8-days day of collection, which implies the number of days after diet implementation, there were no treatment differences within each day. However, the collection days thereafter showed treatment differences within each collection day. By 32 days, 100%RP showed the most depleted δ^13^C (−22.67‰). The 0%RP was enriched from −17.18 to −14.63‰ from 0 to 32 days, while 25%RP remained at −16.53‰ during the same period. Lastly, there were no differences in treatments on δ^15^N composition of the hair (*P* = 0.58; Table 2) and averaged 7.15‰ across treatments.

### Prediction of diet composition

Three subgroups (^13^C fecal 8 days, ^13^C fecal 32 days, and ^15^N fecal 32 days) had concordance correlation coefficients (CCC) ≥ 0.80 (Table 3). They were also characterized by regression model intercepts and slopes relatively close to zero and 1.0, and adjusted R^2^ ≥ 0.70. These subgroups had comparatively favorable profiles with respect to the MSE partitioning (smaller MSE and higher proportion of the MSE due to random error). Several subgroups (^13^C plasma 8 days, ^13^C plasma 32 days, and ^13^C hair 32 days) had Pearson correlation coefficients ≥ 0.80 and regression model adjusted R^2^ ≥ 0.70, but also exhibited low concordance correlation coefficients, model intercepts that deviated substantially from zero, and large MSE with a high proportion attributable to the MC component. Although the predictions for these latter subgroups exhibited strong correlation with the actual dietary percentages, the magnitude of the predictions was meaningfully different from the actual percentages. This translates to not being able to predict dietary percentages directly using Eq. (3).

**Table 3:** Analytic regression analysis results for 16 combinations of isotope (^13^C or ^15^N), day (8 or 32), and sample type (feces, plasma, red blood cell (RBC), or hair).

Isotope	Day	Sample type	Intercept^a^	Slope^a^	Adjusted R^2^	Pearson correlation coefficient	Concordance correlation coefficient (95% BCa bootstrap CI)	Mean squared error^b^	MC^c^	SC^c^	RC^c^
^13^C	8	Fecal	−0.259	0.825	0.84	0.92	0.88 (0.79,0.93)	359.04	33.1%	13.2%	53.7%
^13^C	8	Plasma	60.258	3.988	0.82	0.91	0.13 (0.08,0.18)	3,558.88	77.7%	16.4%	6.0%
^13^C	8	RBC	49.254	−0.998	−0.01	−0.18	−0.02 (−0.09,0.02)	3,950.97	65.2%	4.2%	32.7%
^13^C	8	Hair	51.107	0.577	0.09	0.35	0.12 (0.02,0.49)	3,873.15	69.6%	2.2%	28.2%
^13^C	32	Fecal	−1.666	0.851	0.81	0.90	0.86 (0.76,0.92)	375.27	30.7%	8.4%	60.9%
^13^C	32	Plasma	37.874	1.773	0.94	0.97	0.38 (0.28,0.49)	2,110.74	86.3%	10.4%	3.3%
^13^C	32	RBC	45.830	3.616	0.30	0.58	0.06 (0.02,0.11)	3,438.17	69.4%	6.3%	24.3%
^13^C	32	Hair	27.112	1.318	0.77	0.88	0.51 (0.39,0.67)	1,398.24	76.2%	4.0%	19.8%
^15^N	8	Fecal	13.285	0.543	0.17	0.45	0.39 (−0.06,0.73)	1,487.73	21.0%	12.2%	66.8%
^15^N	8	Plasma	332.172	−1.036	0.15	−0.43	−0.01 (−0.02,−0.00)	51,386.09	96.3%	1.7%	2.9%
^15^N	8	RBC	331.505	−1.308	0.30	−0.58	−0.02 (−0.04,−0.01)	29,412.35	92.8%	4.4%	5.7%
^15^N	8	Hair	79.550	−0.125	0.05	−0.30	−0.03 (−0.17,−0.00)	51,913.15	80.9%	17.0%	2.5%
^15^N	32	Fecal	−3.297	0.933	0.71	0.85	0.83 (0.69,0.92)	406.13	12.9%	1.2%	85.9%
^15^N	32	Plasma	−73.325	0.562	0.01	0.22	0.01 (−0.01,0.02)	29,870.18	95.9%	0.1%	4.0%
^15^N	32	RBC	112.677	−0.279	0.13	−0.40	−0.04 (−0.10,−0.02)	35,909.90	85.2%	11.9%	4.0%
^15^N	32	Hair	58.494	−0.050	0.00	−0.22	−0.04 (−0.09,0.03)	63,761.22	56.4%	41.7%	2.1%

*BCa* bias accelerated and corrected.

^a^From a model regressing actual on predicted dietary proportions. Ideal intercept value is 0.0, ideal slope value is 1.0.

^b^Mean squared error where ‘error’ is the predicted minus actual dietary proportion.

^c^MC, SC, RC are mean, slope, random components of the MSE (as percentages).

The two one-sided test (TOST) with an equivalence region of (−15%,15%) were conducted for the three sets of fecal samples identified above. In each case the mean actual minus predicted difference was < 0 indicating that on average the predicted dietary percentages were greater than the actual percentages (Table 3). The *P*-values for the ^13^C fecal 8 days, ^13^C fecal 32 days, and ^15^N fecal 32 days samples were 0.1035, 0.1039, and 0.0298, respectively. The corresponding minimum equivalence regions for these subgroups were (−16.31, −5.48), (−16.37, −5.11), and (−13.95, −0.51). These results indicate that it would be reasonable to predict dietary percentages directly from Eq. (3) for each of these subgroups with a tolerance of ± 15%.

The analyses where predicted percentages outside of the valid range were assigned the appropriate boundary value prior to analysis resulted in five (one < 0% and four > 100%), three (three > 100%), and two (one < 0% and one > 100%) percentages being reassigned for the ^13^C fecal 8 days, ^13^C fecal 32 days, and ^15^N fecal 32 days subgroups. The results for ^15^N fecal 32 days were essentially unchanged however meaningful improvements were realized for the two ^13^C fecal subgroups with respect to the proximity of the regression slopes to 1.0 (both slopes were very close to the benchmark, although the corresponding intercepts were somewhat farther away from zero than in the primary analyses), the MSE partitioning (smaller MSE with 80% attributable to random error), and the TOST equivalence tests (*P* < 0.01) (Table 4).

**Table 4:** Analytic regression analysis results of predicted vs. actual proportions of rhizoma peanut into bahiagrass hay diets consumed by beef cattle.

Description	δ^13^C				δ^15^N	
	Day 8, fecal without truncation of invalid percentages	Day 8, fecal with truncation of invalid percentages	Day 32, fecal without truncation of invalid percentages	Day 32, fecal with truncation of invalid percentages	Day 32, fecal without truncation of invalid percentages	Day 32, fecal with truncation of invalid percentages
Intercept (ideal value is 0.0)^a^	−0.259	−5.718	−1.666	−9.980	−3.297	−4.708
Slope (ideal value is 1.0)^a^	0.825	0.980	0.851	1.056	0.933	0.956
Adjusted R^2^	0.84	0.84	0.81	0.84	0.71	0.71
Pearson correlation coefficient	0.92	0.92	0.90	0.92	0.85	0.85
Concordance correlation coefficient (95% BCa bootstrap CI)	0.88 (0.79,0.93)	0.90 (0.78,0.96)	0.86 (0.76,0.92)	0.89 (0.75,0.96)	0.83 (0.69,0.92)	0.83 (0.68,0.91)
Mean squared error (MSE)^b^	359.04	241.23	375.27	238.92	406.13	403.33
MC^c^	33.1%	19.5%	30.7%	19.4%	12.9%	13.1%
SC^c^	13.2%	0.2%	8.4%	1.2%	1.2%	0.5%
RC^c^	53.7%	80.3%	60.9%	79.4%	85.9%	86.4%
Mean actual-predicted difference	−10.90	−6.86	−10.74	−6.80	−7.23	−7.26
Equivalence region specified for the TOST test	−15.0, 15.0	−15.0, 15.0	−15.0, 15.0	−15.0, 15.0	−15.0, 15.0	–15.0, 15.0
P-value for the TOST test (Ho: non-equivalence, Ha:equivalence)	0.1035	0.0043	0.1039	0.0040	0.0298	0.0298
Minimum equivalence region required to declare equivalence at the 0.05 level	−16.31, −5.48	−11.72, −1.99	−16.37, −5.11	− 11.65, −1.96	−13.95, −0.51	−13.95, −0.57

Fecal samples were analyzed for δ^13^C and δ^15^N and diets were reconstructed using two-pool mixing models. Truncated values indicate predicted values re-assigned appropriate boundary value, where predicted proportions of < 0% were assigned a value of 0% and those > 100% were assigned a value of 100%.

^a^From a model regressing actual on predicted dietary proportions**.**

^b^Mean squared error where 'error' is the predicted minus actual dietary proportion**.**

^c^MC, SC, RC are mean, slope, random components of the MSE (as percentages), respectively.

## Discussion

The various samples collected in this study differed in their δ^13^C and δ^15^N compositions throughout the 32-days period, and therefore their utility for use as predictor of diet composition varies. The use of δ^13^C across the samples collected proved a more reliable predictor, than δ^15^N, of C_3_–C_4_ diet composition. However, the δ^15^N of feces indicated a probable method for estimating C_3_:C_4_ diet composition, which was unexpected. Additionally, the response to δ^13^C composition was also affected by the collection day, implying the time after diet change affects these results and reliability of these samples as C_3_–C_4_ diet predictors.

Altogether, the fecal δ^13^C results corroborate data by Norman, et al. ^6^, where feces from sheep fed various proportions of C_3_–C_4_ forages remained at δ^13^C equilibrium within 18-days after dietary change and were useful predictors of diet composition. Rates of passage will have an impact on fecal composition, and therefore the isotopic composition of the fecal material is reflective of the ingested diet after the retention period along the digestive tract. Bahiagrass has a mean retention time of 42 h in rumen^15^, suggesting that feces will likely reflect dietary changes beyond this time period, especially as the animal integrates the diet into their system^16^. In addition, considering total tract retention of diet feedstuff is important for ensuring accuracy of sample collection. Therefore, the fecal δ^13^C obtained 8-days after diet change, does indeed represent the diet consumed within this 8-days time frame, and given that fecal sample collection does not require specialized equipment, additional personnel training, or elaborate sample processing, this methodology will likely continue to be favored over the collection and analysis of other samples.

Regarding fecal δ^15^N, enrichment (in relation to the diet) has also been observed in other studies, and it is likely a result of ^15^N discrimination during the digestive process^17–19^. Sutoh, et al. ^17^ also showed rumen bacteria and protozoa differ in their δ^15^N composition, which might also provide insights into the δ^15^N metabolic fates. In meerkat (*Suricata* *suricatta*) feces, δ^15^N enrichment occurred during transit through the digestive system, indicating discrimination is not restricted to ruminant species alone^19^. Discrimination factors (Δ) are likely affected by the diet composition^19^. For instance, Sare, et al. ^20^ established that δ^15^N composition in stomach contents, feces, collagen, and hair collected from red-backed voles (*Clethrionomys* *gapperi*) varied in each of the sample types according to the protein concentration in the diets. Similar studies are warranted in ruminants, especially to establish accurate discrimination factors since models have been developed that incorporate diet-sample discrimination^6^.

In this study, fecal δ^15^N was useful for predicting diet proportions at 32-days, owed to the strong CCC and adequate adjusted R^2^ from regression of actual vs. observed proportions of RP in diet (Table 3). These results are novel since previous uses of ^15^N have not resulted in capability of predicting dietary proportions, especially that of C_3_–C_4_ diets. Possible explanation may be attributed to the magnitude in difference of the feed δ^15^N in 0%RP and 100%RP diets, which was 1.92‰, was large enough such that differences in species composition could be detected, similar to the mechanisms observed using δ^13^C. However, Cantalapiedra-Hijar et al. ^21^ indicate that differences in animal ^15^N discrimination are highly affected by the animal’s metabolism. While fecal δ^15^N may be used as a diet predictor in C_3_–C_4_ binary mixtures, further studies are still warranted to corroborate these results and evaluate if differences may occur due to an animal’s age or stage of production. If corroborated, these results may provide options for potentially reconstruction diet components in diets consisting only of either C_3_ or C_4_ forages, so long as the feed δ^15^N differences are large in magnitude.

The time required to reach equilibrium is directly influenced by the range in isotopic compositions of the diets consumed, and is also affected by the magnitude in change of diet isotopic composition^6^. In plasma there is a high correlation at both 8 and 32-d for predicted vs. observed RP diet proportions, however the intercept of the model falls below any realistic value, along with poor CCC, which results in predictions well below 0% across all treatments (Table 2). It may be speculated that a period beyond 32-d may be required to establish plasma δ^13^C equilibrium. Similar results are reported to other species, including American Black Ducks (*Anas* *rubripes*), where diet changes were detected using δ^13^C of plasma within 1 week after diet change, but required at least 67 days to reach δ^13^C equilibrium ^22^.

Isotopic fractionation in plasma N has been reported in other studies and is attributed to liver urea synthesis and splanchnic amino acid metabolism ^17,23^. In addition, fractionation in plasma N can also be affected by both diet type and nutrient intake level^23^. Plasma δ^15^N might be a better indicator of the N status of the animal by encompassing variability in the conversion of metabolizable N into animal proteins ^23^. Halley et al. ^24^ show that ^15^N fractionation in male reindeer (*Rangifer* *tarandus*) varies by individuals, tissue/sample collected, sex, and even physiological status of the animal, thus establishing accurate fractionation values is important for increasing their value in diet-reconstruction models. In the present study, the observed plasma δ^15^N did not appear to reach equilibrium, since each of the collection days differed in their δ^15^N composition and the overall values appeared to become depleted with time. The inherent variability in body tissues and within animal species, as well as differences in turnover rates may affect the predictive power across different body samples collected^25^. Results should be interpreted with caution, especially when adequate equilibrium periods have not been established for animals on a specific diet ^23,26^.

The utility of RBC as diet component indicator was limited for the duration of this study. Bovine RBC lifespan is relatively long, between 130 and 160 d, indicating that the isotopic composition in RBC may not reach an equilibrium until this time period ^27–29^, therefore an experiment extending beyond 160 d may be more representative of RBCs produced from nutrients absorbed from the diets provided. In essence, RBC δ^13^C composition is more greatly influenced by what the animal has consumed throughout a longer time frame ^30^. Other potential uses of RBC δ^13^C or δ^15^N compositions include the capability of estimating foraging and migratory behavior of waterfowl^22^ or other wildlife^24^.

Stable isotope analyses of keratinized tissues, such as hair, have been useful for dietary reconstruction of ruminant diets ^31^, as well as other species^32^. Zazzo, et al. ^31^ also detected dietary changes through analysis of hair in beef steers consuming C_3_ or C_4_ forage-based diets. The use of keratinized tissues offers several advantages since keratin generally contains all the major light elements (C,H, N, O, and S) and can be sampled non-invasively ^31^. Hair growth rates and length will pose an inherent challenge to the accurate representation of the animal’s diet, especially since these vary between breed and individuals ^31^. For the purposes of this study, new growth was always collected since all animals were shaved from the same location at each evaluation period. Even though by 32 d there was greater correlation between observed and predicted RP proportions, the CCC remained low, indicating the values predicted were still less than the observed. This might indicate the dietary history of the steers through a wider period of time, rather than being indicative of more recent dietary composition. Hair samples may contain dietary information for up to 15 mo., though further studies are required to establish adequate equilibration periods, as well as to establish differences among breeds ^31,33^.

In conclusion, the accurate establishment of isotopic equilibrium across body samples is still required to assure accuracy of utilizing stable isotopes for predicting diet composition of cattle consuming C_3_–C_4_ binary mixed diets. Each sample type has contrasting equilibrium times, which in turn, may reflect relative diet composition across different time scales (e.g. days, weeks, or months etc.). Estimating diet intake proportions in the short term (e.g. 8 days after diet change) was best utilizing fecal δ^13^C, compared to all other sample types evaluated. In the longer term (e.g. 32-days after diet change), feces δ^13^C and δ^15^N were both considered adequate predictors of diet composition. Despite plasma and hair showing high correlation between predicted and actual values, the estimates were not adequate enough to provide accurate predictions of diet composition in C_3_–C_4_ binary mixtures at 32-days after diet change. The nature of protein turnover and deposition in plasma and hair requires a larger window of time before they represent the animal’s diet. Provided that fecal sample collection is non-invasive, does not require additional processing equipment, or trained personnel, it is thus concluded that fecal δ^13^C continues to be the optimal method to estimate diet proportions in C_3_–C_4_ binary diets. Further evaluation into the usefulness of fecal δ^15^N for diet prediction is warranted, as this has not been yet evaluated in beef cattle.

## Materials and methods

### Animals, housing, and treatments

All procedures involving animals were approved by the University of Florida Institutional Animal Care and Use Committee (Protocol #201709925). All methods were performance in accordance with the relevant guidelines and regulations, and permission and informed consent was obtained from the University of Florida (owners) for the use of the steers in this experiment.

The experiment was carried out during July and August of 2017 at the Feed Efficiency Facility of University of Florida, North Florida Research and Education Center, located in Marianna, Florida (30°52′N, 85°11″W, 35 m asl). Both ‘Argentine’ bahiagrass and ‘Florigraze’ rhizoma peanut hays were obtained from commercial producers. The hay bales were stored in enclosed barns throughout the duration of the experiment.

Twenty-five Brahman × Angus crossbred steers (*Bos* sp.) were utilized (average BW = 341 ± 17 kg, approx. 16 months of age). The steers were grazing bermudagrass (*Cynodon* *dactylon*) pastures, a C_4_ grass, prior to the start of the study. The day prior to the start of the experiment (e.g. day-1), steers brought to working facilities, where they remained 16 h off feed and water, in order to obtain shrunk bodyweights. On day 0 of the experiment, steers were weighed, blocked by bodyweight, and allocated to five treatments (5 steers per treatment) and housed in grouped pens. Hay intake was recorded utilizing GrowSafe^©^ systems (GrowSafe Systems Ltd., Calgary, AB, Canada), which utilize radio frequency identification to record feed intake by weight change measured to the nearest gram. Water was available ad libitum. Forage treatments were offered ad libitum by providing sufficient hay to maintain full feed troughs throughout each day of the experiment. Treatments were five proportions of ‘Florigraze’ rhizoma peanut hay in ‘Argentine’ bahiagrass hay: (1) 100% bahiagrass hay (0% RP); (2) 25% rhizoma peanut hay + 75% bahiagrass hay (25% RP); (3) 50% rhizoma peanut hay + 50% bahiagrass hay (50% RP); (4) 75% rhizoma peanut hay + 25% bahiagrass hay (75% RP); (5) 100% rhizoma peanut hay (100% RP). Diet chemical composition is presented in Table 1. All treatment proportions were weighed and mixed on as-fed basis. Mixing of diets was done manually; no hay mixers or choppers were used, to minimize leaf shatter.

### Sample collection

Steers were housed for 32 days and sampling occurred on 0, 8, 16, 24, and 32 days after initiation of treatment diets; exception was for feces, which were collected on d-1 given steers were fasted on d-0 of the experiment. The hay mixtures offered to the steers were collected (10 samples of each diet) and analyzed for nutritive value (Table 1), at the start of the experiment. All sampling occurred between 0700 and 1000 h on each of the sampling days.

Fecal samples were collected directly from the rectum and placed in quart-sized plastic bags to avoid contamination. The feces were frozen at −20 °C. All fecal samples were thawed, dried at 55 °C for 72 h, and ground to pass a 2-mm stainless steel screen using a Wiley Mill (Model 4, Thomas-Wiley Laboratory Mill, Thomas Scientific, Swedesboro, NJ, USA). Samples were then ball milled using a Mixer Mill MM400 (Retsch GmbH, Haan, Germany) at 25 Hz for 9 min.

Blood was obtained through jugular venipuncture using 10-mL K_2_ EDTA vials (Becton Dickinson and Company, Franklin Lakes, NJ, USA), and stored in ice until centrifugation. All tubes were centrifuged at 714 G for 20 min using an Allegra X-22R centrifuge (Beckman Coulter, Brea, CA, USA). A 10-mL sample of plasma was collected and placed in a separate glass vial, the remaining plasma, white blood cell, and platelet fractions were discarded. The remaining RBC pellet was re-suspended with 9 vol. 0.9% NaCl solution and mixed at room temperature for 15 min at 2 Hz orbital shaker. The tubes were then centrifuged at 714 G for 20 min. The saline solution from the centrifuged tubes was discarded after centrifugation. The rinse procedure was repeated two more times for a total of three rinses. After the third rinse procedure, a 500-µL sample was removed, frozen at −20 °C, and subsequently freeze-dried for isotopic analyses.

Hair clippings were obtained from an area of 20 × 20 cm on the left hindquarter, utilizing veterinary hair clippers (Sunbeam-Oster Inc., Boca Raton, FL, USA). Hair clippings were collected, placed in nylon bags (Ankom Technology, Macedon, NY, USA), and frozen for subsequent analysis. Clippings were always collected in the same location from each animal in order to ensure new hair growth would be analyzed. All hair samples were cleaned using soapy water and defatted following protocols for other keratin-based tissues ^31,34^. Each sample was sonicated twice for 30 min in a methanol and chloroform solution (2:1, v/v), rinsed with distilled water, and oven dried overnight at 60 °C. Each hair sample was ball milled using a Mixer Mill MM400 (Retsch GmbH, Haan, Germany) at 25 Hz for 9 min.

### Calculations

After processing, all samples were analyzed for total C and N using a CHNS analyzer through the Dumas dry combustion method (Vario MicroCube, Elementar Americas Inc., Ronkonkoma, NY, USA) coupled to an isotope ratio mass spectrometer (IsoPrime 100, Elementar, Elementar Americas Inc., Ronkonkoma, NY, USA). Certified standards of L-glutamic acid (USGS40, USGS41; United States Geological Survey) were used for isotope ratio mass spectrometer calibration. Isotope ratios were as follows: δ^13^C of −26.39, + 37.63‰, and δ^15^N of −4.52, 47.57‰ for USGS40 and USGS41, respectively. Calibration of the IRMS was conducted according to Cook, et al. ^35^, with an accuracy of ≤ 0.06 ‰ for ^15^N and ≤ 0.08 ‰ for ^13^C.

The isotope ratio for ^13^C/^12^C was calculated as:

1 $$\delta^{{{13}}} {\text{C}} = \, \left( {^{{{13}}} {\text{C}}/^{{{12}}} {\text{C}}_{{{\text{sample}}}} {-}^{{{13}}} {\text{C}}/^{{{12}}} {\text{C}}_{{{\text{reference}}}} } \right)/ \, \left( {^{{{13}}} {\text{C}}/^{{{12}}} {\text{C}}_{{{\text{reference}}}} \times { 1}000} \right)$$

where δ^13^C is the C isotope ratio of the sample relative to Pee Dee Belemnite (PDB) standard (‰), ^13^C/^12^C_sample_ is the C isotope ratio of the sample, and ^13^C/^12^C_reference_ is the C isotope ratio of PDB standard ^5^. The isotope ratio for ^15^N/^14^N was calculated as:

2 $$\delta^{{{15}}} {\text{N}} = \, \left( {^{{{15}}} {\text{N}}/^{{{14}}} {\text{N}}_{{{\text{sample}}}} -^{{{15}}} {\text{N}}/^{{{14}}} {\text{N}}_{{{\text{reference}}}} } \right)/\left( {^{{{15}}} {\text{N}}/^{{{14}}} {\text{N}}_{{{\text{reference}}}} \times { 1}000} \right)$$

where δ^15^N is the N isotope ratio of the sample relative to atmospheric nitrogen (‰), ^15^N/^14^N_sample_ is the N isotope ratio of the sample, and ^15^N/^14^N_reference_ is the N isotope ratio of atmospheric N (standard) ^5^. The fraction factor (Δ) in this study was considered to be the difference between the diet isotopic composition (δ^13^C or δ^15^N) and that of the given sample ^5^.

The dietary proportion of rhizoma peanut hay was back-calculated using δ^13^C and δ^15^N of each plant on a DM basis ^3^. This method is advantageous in that it does not require further tissue processing and facilitates implementation at the field scale. The proportion of rhizoma peanut was estimated using Eq. (3), described by Jones et al. ^3^:

3 $$\%RP=100-\left\{100 \times \frac{A-C}{B-C}\right\}$$

where %RP is the proportion of RP in the diet, *A* is the δ^13^C or δ^15^N of the sample obtained, *B* is the δ^13^C or δ^15^N of bahiagrass, and *C* is the δ^13^C or δ^15^N of RP.

### Statistical analysis

All response variables were analyzed using linear mixed model procedures as implemented in SAS PROC GLIMMIX (SAS/STAT 15.1, SAS Institute). Individual animals were considered the experimental unit. Treatment, collection day, and their interaction were considered fixed effects, and block was considered a random effect in the model. The data were analyzed as repeated measures, considering collection day as the repeated measure. The best covariance matrix was selected according to the lowest AICC fit statistic. Least squares treatment means were compared through pairwise *t* test using the PDIFF option of the LSMEAN statement in the aforementioned procedure. Based on the recommendations by Milliken and Johnson ^36^ and Saville ^37^, no adjustment for multiple comparisons was made. Orthogonal polynomial contrasts (linear and quadratic effects) were used to test effects of RP inclusion on isotopic responses. The slice option was used when the treatment × collection day interaction was significant (*P* ≤ 0.05), using collection day as the factor, to test treatment effects across collection days. Significance was declared at *P* ≤ 0.05. The contrast statement was used to test for linear or quadratic effects. Regression analyses for the dietary predictions were conducted using PROC REG from SAS.

Predictions of dietary proportions based on Eq. (3) were generated for 16 subgroups reflecting combinations of isotope (^13^C or ^15^N), day (8 or 32), and sample type (feces, plasm, RBC, or hair). Analyses comparing predicted versus actual diet proportions included several components. First, we computed the concordance correlation coefficient (CCC) following the recommendations from Crawford, et al. ^38^. The CCC is a measure of agreement that encompasses both precision and accuracy, along with corresponding 95% bias accelerated and corrected (BCa) bootstrap confidence intervals. For comparative purposes we calculated the Pearson correlation coefficient which only reflects precision. Both correlation coefficients range from −1.0 to 1.0 and we interpreted values ≥ 0.80 as indicating strong positive agreement/correlation. Next, we regressed the actual percentages on the predicted percentages using linear regression. Perfect prediction corresponds to the estimated regression line having an intercept of zero and a slope of 1.0. We then partitioned the mean square error (MSE) of the predicted (from Eq. (3), not the above linear regression) versus actual percentages as described in Rice and Cochran ^39^. This partitioning entails calculating the proportion of MSE attributable to three sources of error: the difference in mean predicted and actual values (mean component, denoted “MC”), the error resulting from the slope of the above linear regression deviating from 1.0 (slope component, denoted “SC”), and random error (random component, denoted “RC”). The results from the above analyses were examined to identify subgroups whose predictions were sufficiently good to warrant hypothesis testing. In this context “good” means that the predicted percentages were strongly correlated with the actual percentages and the magnitudes of the predicted percentages were similar to the actual percentages. The objective of the hypothesis testing was to formally evaluate whether dietary proportions could be directly predicted from Eq. (3) (in contrast to generating predictions using the equation from regressing actual dietary percentages on the predicted percentages from Eq. (3)). Paired two one-sided test (TOST) equivalence tests were conducted for the selected subgroups with α = 0.05^40^. These tests are formulated such that the null hypothesis is “non-equivalence” and the alternative hypothesis is “equivalence”. An input parameter to the test is the equivalence region, a range for which we consider the mean actual minus predicted difference to be unimportant (“equivalent”) from a practical standpoint. For each equivalence test we also computed the 90% confidence interval for the mean actual minus predicted difference which we refer to as the “minimum equivalence region”. Based on the structure of TOST equivalence tests, to reject the null hypothesis at the 0.05 level, the equivalence region specified for the test must completely contain the minimum equivalence region. For example, if the pre-specified equivalence region is (−15%, 15%) and the computed minimum equivalence region is (−16%, −6%) the null hypothesis would not be rejected. Finally, we re-ran all of the analyses described above for the selected subgroups where, prior to analysis, predicted percentages outside of the valid range were assigned the appropriate boundary value (i.e., predicted percentages < 0% were assigned a value of 0% and those > 100% were assigned a value of 100%).

## Data Availability

The datasets used and/or analyzed during the current study are available from the corresponding authors on a reasonable request.

## References

[1] Gregorini P, Villalba JJ, Provenza FD, Beukes PC, Forbes JM (2015). Modelling preference and diet selection patterns by grazing ruminants: A development in a mechanistic model of a grazing dairy cow. MINDY Anim. Prod. Sci..

[2] Allen VG (2011). An international terminology for grazing lands and grazing animals. Grass Forage Sci..

[3] Jones RJ, Ludlow MM, Troughton JH, Blunt CG (1979). Estimation of the proportion of C3 and C4 plant species in the diet of animals from the ratio of natural ^12^C and ^13^C isotopes in the faeces. J. Agric. Sci..

[4] Dove H, Mayes RW (1996). Plant wax components: A new approach to estimating intake and diet composition in herbivores. J. Nutr..

[5] Fry B (2006). Stable Isotope Ecology.

[6] Norman HC, Wilmot MG, Thomas DT, Masters DG, Revell DK (2009). Stable carbon isotopes accurately predict diet selection by sheep fed mixtures of C3 annual pastures and saltbush or C4 perennial grasses. Livest. Sci..

[7] Farquhar GD, Ehleringer JR, Hubick KT (1989). Carbon isotope discrimination and photosynthesis. Annu. Rev. Plant Biol..

[8] Unkovich M (2008). Measuring Plant-Associated Nitrogen Fixation in Agricultural Systems.

[9] Shearer G, Kohl DH (1986). N2-fixation in field settings: Estimations based on natural ^15^N abundance. Funct. Plant Biol..

[10] Peterson BJ, Fry B (1987). Stable isotopes in ecosystem studies. Annu. Rev. Ecol. Syst..

[11] Boddey, R. M. & Dobereiner, J. Nitrogen economy in tropical soils*.* in *Proceedings**of**the**International**Symposium**on**Nitrogen**Economy**in**Tropical**Soils,**Held**in**Trinidad,**W.I.,**January**9–14,**1994* (Ahmad, N. ed.). 241–250 (Springer Netherlands, 1996).

[12] Jensen ES (2012). Legumes for mitigation of climate change and the provision of feedstock for biofuels and biorefineries: A review. Agron. Sustain. Dev..

[13] Santos ERS (2019). Sward responses of Bahiagrass cultivars under no nitrogen fertilization. Crop Sci..

[14] Pereira Neto JD (2019). Tracing back sheep diet composition feeding grass-legume mixtures using fecal δ^13^C. Small Ruminant Res..

[15] Flores J, Moore J, Sollenberg L (1993). Determinants of forage quality in Pensacola bahiagrass and Mott elephantgrass. J. Anim. Sci..

[16] Sponheimer M, Robinson T, Ayliffe L, Passey B (2003). An experimental study of carbon-isotope fractionation between diet, hair, and feces of mammalian herbivores. Can. J. Zool..

[17] Sutoh M, Koyama T, Yoneyama T (1987). Variations of natural ^15^N abundances in the tissues and digesta of domestic animals. Radioisotopes.

[18] Cheng L, Kim EJ, Merry RJ, Dewhurst RJ (2011). Nitrogen partitioning and isotopic fractionation in dairy cows consuming diets based on a range of contrasting forages. J. Dairy Sci..

[19] Montanari S (2017). Discrimination factors of carbon and nitrogen stable isotopes in meerkat feces. PeerJ.

[20] Sare DTJ, Millar JS, Longstaffe FJ (2005). Tracing dietary protein in red-backed voles (*Clethrionomys*
*gapperi*) using stable isotopes of nitrogen and carbon. Can. J. Zool..

[21] Cantalapiedra-Hijar G (2016). Relationship between efficiency of nitrogen utilization and isotopic nitrogen fractionation in dairy cows: Contribution of digestion v. metabolism?. Animal.

[22] Barboza PS, Jorde DG (2018). Monitoring responses to variation in food supply for a migratory waterfowl: American Black Duck (*Anas*
*rubripes*) in winter. J. Comp. Physiol. B Biochem. Syst. Environ. Physiol..

[23] Cantalapiedra-Hijar G (2015). Diet-animal fractionation of nitrogen stable isotopes reflects the efficiency of nitrogen assimilation in ruminants. Br. J. Nutr..

[24] Halley DJ, Minagawa M, Nieminen M, Gaare E (2010). Diet: tissue stable isotope fractionation of carbon and nitrogen in blood plasma and whole blood of male reindeer *Rangifer*
*tarandus*. Polar Biol..

[25] Kurle CM (2002). Stable-isotope ratios of blood components from captive northern fur seals (*Callorhinus*
*ursinus*) and their diet: Applications for studying the foraging ecology of wild otariids. Can. J. Zool..

[26] Gannes LZ, Del Rio CM, Koch P (1998). Natural abundance variations in stable isotopes and their potential uses in animal physiological ecology. Comp. Biochem. Physiol. A Mol. Integr. Physiol..

[27] Wood, D. & Quiriz-Rocha, G. *Schalm's**Veterinary**Hematology* (ed. Wardrop, D.J., Weiss, K.J.). 829–835 (Wiley, 2010).

[28] Brockus, C. W. *Duncan**and**Prasse's**Veterinary**Laboratory**Medicine:**Clinical**Pathology* (ed. Latimer, K.S.). 3–44 (2011).

[29] Roland L, Drillich M, Iwersen M (2014). Hematology as a diagnostic tool in bovine medicine. J. Vet. Diagn. Invest..

[30] Zeppelin TK, Johnson DS, Kuhn CE, Iverson SJ, Ream RR (2015). Stable isotope models predict foraging habitat of northern fur seals (*Callorhinus*
*ursinus*) in Alaska. PLoS ONE.

[31] Zazzo A (2007). Experimental determination of dietary carbon turnover in bovine hair and hoof. Can. J. Zool..

[32] Nakashita R, Hamada Y, Hirasaki E, Suzuki J, Oi T (2013). Characteristics of stable isotope signature of diet in tissues of captive Japanese macaques as revealed by controlled feeding. Primates.

[33] French MH (1946). Growth rates of hair on grade European and indigenous breeds of cattle. East Afr. Agric. J..

[34] O'Connell TC, Hedges RE, Healey M, Simpson AHR (2001). Isotopic comparison of hair, nail and bone: Modern analyses. J. Archaeol. Sci..

[35] Cook CS (2017). Stable Isotope Biogeochemistry and Ecology: Laboratory Manual.

[36] Milliken GA, Johnson DE (2009). Analysis of Messy Data.

[37] Saville DJ (2015). Multiple comparison procedures-cutting the gordian knot. Agron. J..

[38] Crawford SB, Kosinski AS, Lin HM, Williamson JM, Barnhart HX (2007). Computer programs for the concordance correlation coefficient. Comput. Methods Programs Biomed..

[39] Rice JA, Cochran PA (1984). Independent evaluation of a bioenergetics model for largemouth bass. Ecology.

[40] Schuirmann DJ (1987). A comparison of the two one-sided tests procedure and the power approach for assessing the equivalence of average bioavailability. J. Pharmacokinet. Biopharm..

[41] Moore JE, Mott G (1974). Recovery of residual organic matter from in vitro digestion of forages. J. Dairy Sci..

